# Acute Psychosis After Treatment of Epididymitis with Ciprofloxacin

**DOI:** 10.7759/cureus.2605

**Published:** 2018-05-10

**Authors:** Garrett Rossi, Kristen Mazoki

**Affiliations:** 1 Psychiatry, Cooper University Hospital, Camden, USA

**Keywords:** acute psychosis, epididymitis, ciprofloxacin, infectious diseases, psychiatry

## Abstract

Ciprofloxacin is a broad-spectrum antibiotic that is generally well-tolerated and widely used in clinical practice to treat various forms of infection. There have been several case reports detailing the development of psychosis and other neuropsychiatric symptoms secondary to use of this antibiotic. This case report details the history of an acute paranoid psychosis that developed in a patient treated with ciprofloxacin for epididymitis.

## Introduction

Ciprofloxacin is a broad-spectrum antibiotic of the fluoroquinolone group that is generally well-tolerated. Ciprofloxacin is active against both gram-negative and gram-positive bacteria [1]. It functions by inhibiting deoxyribonucleic acid (DNA) gyrase, type II topoisomerase, and topoisomerase IV, which are required to separate bacterial DNA, thus inhibiting cell division [1]. The most commonly reported drug-related adverse events from clinical trials of all formulations, dosages, and therapy durations were nausea, diarrhea, abnormal liver function tests, vomiting, and rash. In 2013, the Food and Drug Administration (FDA) warned of potential central nervous system side effects related to the use of ciprofloxacin and other fluoroquinolones [2]. The most common central nervous system (CNS) effects include headache, dizziness, and insomnia [2]. More serious neuropsychiatric side effects, including psychosis, anxiety, hallucinations, paranoia, and suicide attempts, have been observed [2].

## Case presentation

The patient is a 36-year-old African American male who initially presented to the emergency department with a chief complaint of feeling confused for the past day. He also complained of depression, anxiety, and paranoia. The patient declined psychiatric evaluation and medical admission at that time and was discharged home. The patient was urged to come back to the hospital by his wife and mother approximately 14 hours later due to disorientation, confusion, and agitation. At that time, he was admitted for an altered mental status workup with the working diagnosis of medication-induced delirium. Psychiatry was consulted to the medical floor and one-to-one observation was recommended. The patient underwent a full neurologic workup, including a computed tomography (CT) scan of the head and a lumbar puncture, which were unremarkable. The patient elected to leave against medical advice after two days. He presented to the emergency department two days later with similar complaints. At that time, all labs, including urinalysis, complete metabolic panel, urine drug screen, complete blood count, and vital signs, were within normal limits, and he was medically cleared and evaluated by psychiatry in the emergency department. 

During this evaluation, the patient was calm and cooperative. He reported being diagnosed with a “testicular infection” and was prescribed a course of ciprofloxacin, 500 mg for 10 days. He reported that he was not thinking clearly after starting the antibiotic and stated that he had “some side effects” but was too paranoid to elaborate. The medication was discontinued during his previous admission. He was unable to provide any details regarding the reason for admission and stated that it was his wife’s idea to come to the hospital. The patient appeared internally preoccupied, guarded, and paranoid throughout the evaluation process. He denied any auditory or visual hallucinations. The patient once again requested to sign out of the hospital against medical advice. The patient was paranoid about what the medical staff was doing with his blood and stated he did not feel safe in the hospital. Collateral information was obtained from the patient’s wife who reported that the patient's behavior changed abruptly after starting the ciprofloxacin. She reported that the patient was very paranoid and accused her of attempting to poison him. 

The patient had no previous psychiatric history. The patient reported marijuana use since age 17. He recently stopped using marijuana two weeks prior to treatment for the diagnosis of epididymitis; he had used approximately one gram every other day prior to stopping. Social history revealed the patient is married with three children and employed as a construction worker. The patient denied any previous medical or surgical history. 

The differential diagnosis included medication-induced delirium, drug-induced psychosis, cannabis-induced psychotic disorder, and brief psychotic disorder. The patient was admitted to the psychiatric inpatient unit. After two days of hospitalization and five days after discontinuing ciprofloxacin, the patient’s paranoia resolved and he was discharged to outpatient follow-up. 

## Discussion

Ciprofloxacin is a widely used antimicrobial agent that is routinely prescribed in clinical practice. There are very few cases of psychosis secondary to ciprofloxacin reported in the literature [3-9]. The total incidence of adverse effects is estimated as 9.3% [10]. The common adverse events include nausea, diarrhea, abnormal liver function tests, vomiting, and rash [10]. The most common side effects are headache, dizziness, and insomnia [2]. Documented central nervous system side effects include convulsions, catatonia, and psychotic reactions. The patient had no past psychiatric history, had a normal metabolic screen, no exposure to other medications known to induce psychosis, and a documented history of recent exposure to ciprofloxacin. The patient’s symptoms began with exposure to the antibiotic and ended when the patient stopped the antibiotic. It is difficult to determine with certainty if exposure to ciprofloxacin was the cause of the patient’s symptoms as re-exposure would be the only way to definitively determine this. 

The mechanism by which ciprofloxacin and fluoroquinolones induce neuropsychiatric symptoms is unclear. Some of the papers uncovered during the literature search revealed potential mechanisms of action. It is believed that the antibiotic may inhibit binding of gamma-aminobutyric acid (GABA) in the brain at the GABA_A_ receptors, leading to upregulation of glutamatergic neurotransmission [9]. Drugs known to induce psychosis, such as ketamine and phencyclidine, do so via upregulation of glutamatergic neurotransmission [9]. It is further suggested that the neuropsychiatric side effects are dose-dependent, and thus antibiotic concentration and dosing parameters should be carefully evaluated [11]. More research needs to be conducted regarding the effects of the strength of dose and timing of the administration of ciprofloxacin to definitively say what effect these factors have on the development of psychosis.

## Conclusions

Various side effects of fluoroquinolones are well-documented in the literature. While psychosis is a rare and an unusual side effect, it’s important to keep drug-induced psychosis in mind as a possible differential. It’s important for the consultation psychiatrist to consider this when evaluating patients who present with acute onset psychosis, no previous psychiatric history, and recent exposure to fluoroquinolones.
